# Trends in the prevalence of childhood allergic diseases in Japan: Comparison of surveys conducted in 1982, 1992, 2002, 2012, and 2022 (WJSAAC phase I–V)^☆^

**DOI:** 10.1016/j.waojou.2026.101396

**Published:** 2026-05-14

**Authors:** Sankei Nishima, Hiroshi Odajima, Hiroshi Matsuzaki, Junichiro Tezuka

**Affiliations:** a Department of Pediatrics, NHO Fukuoka National Hospital, Fukuoka, Japan; b Department of Allergy and Pulmonology, Fukuoka Children's Hospital, Fukuoka, Japan

**Keywords:** Allergic diseases, Allergic rhinitis, Allergic conjunctivitis, Japanese cedar pollinosis, Prevalence

## Abstract

**Background:**

The prevalence of allergic diseases is increasing worldwide. This study aimed to investigate the prevalence of asthma and other allergic diseases among elementary school children in western Japan, as well as associations with age, gender, family history, a history of respiratory infections in early childhood, and residential area, etc., based on 5 surveys conducted between 1982 and 2022.

**Methods:**

Using a consistent methodology, we assessed the prevalence of allergic diseases among children aged 6–12 years in 5 surveys. The surveys were conducted in 1982, 1992, 2002, 2012, and 2022. Allergic diseases other than bronchial asthma (BA) were investigated from 1992 onward.

**Results:**

The number of subjects analyzed was 29,553—down from 55,388. The prevalence of atopic dermatitis (AD) decreased from 17.3% to 13.0%. Meanwhile, allergic rhinitis (AR) increased from 15.9% to 33.9%, allergic conjunctivitis (AC) from 6.7% to 12.2%, and Japanese cedar pollinosis (JCP) from 3.6% to 14.4%. The combined prevalence of AR, AD, and bronchial asthma (BA) increased from 29.4% in 1992 to 37.8% in 2022. The prevalence of AR, AC, and JCP increased significantly, especially among older children, while the prevalence of AD and BA decreased. With the exception of AD, boys generally tended to be more affected than girls. A family history of allergic disease and a history of childhood respiratory infections were important risk factors.

**Conclusion:**

Trends in the prevalence of allergic diseases vary by disease, and long-term studies using the same geographical area and methods, including investigation of confounding factors, are needed.

## Introduction

An increase in allergic diseases has been reported globally, leading to the establishment of the International Study of Asthma and Allergies in Childhood (ISAAC) to understand its prevalence and causes. Phase I trials began in 1995, followed by Phase II trials, and Phase III trials were conducted from 2001 to 2002.^1^, ^2^, ^3^, ^4^ Japan participated in the Phase I and Phase III trials, and part of the Phase II trials. In western Japan, the first prevalence survey of bronchial asthma (BA) was conducted in 1982, targeting 55,388 elementary school children across 11 prefectures (West Japan Study of Asthma and Allergies in Childhood: WJSAAC-I).^5^ Subsequent surveys were conducted in the same area in 1992 and 2002 to investigate the prevalence of allergic diseases by adding atopic dermatitis (AD), allergic rhinitis (AR), allergic conjunctivitis (AC), and Japanese cedar pollinosis (JCP).^6^ In 2012 (WJSAAC-IV) and 2022 (WJSAAC-V), the study for food allergy (FA) and anaphylaxis (An) were added to the survey. BA has been comprehensively reported elsewhere based on the same WJSAAC survey series. Therefore, the present study focuses primarily on long-term trends in other major allergic diseases, while BA is described only as needed to provide an integrated understanding of temporal changes in the overall allergic disease spectrum. In this report, we compared and analyzed the results of 4 surveys, WJSAAC∸II to WJSAAC∸V, for 4 diseases: AR, AD, AC, and JCP.

There are very few large-scale epidemiological studies using the same methods and in the same area over a 40-year period, so the aim of this paper is that we report these results as basic data on future study of allergic disease prevalence rates.

## Subjects and methods

The subjects were 55,388 to 29,553 elementary school children aged 6–12 years from 11 prefectures in western Japan.

The survey began in 1982 with the first phase of asthma surveillance and has been conducted every 10 years since then. In 1992, surveys of AD, AR, AC, and JCP were added.

Here we report the results of the surveys on allergic diseases conducted in phase II to V. The questionnaire adhered to the American Thoracic Society-Division of Lung Diseases (ATS-DLD) Japanese version and its revised version for BA, and wheeze (W), as in the Phase I trial (1982). The definitions for the diagnoses of these diseases in the questionnaire are the same as previously reported^1^^,^^7^ as follows;

**AD** was defined as a positive response to both of the following questionnaire items: (1) “Have you ever been told by a doctor that you have eczema or atopic dermatitis?” and (2) “Does it still occur now?”

**AR** was defined as a positive response to both of the following questionnaire items: (1) “Have you ever been diagnosed with allergic rhinitis or hay fever?” and (2) either “Do you currently have rhinitis symptoms (sneezing, runny nose, nasal congestion, etc.)?” or “Are these symptoms particularly severe from February to April?”

**AC** was defined as a positive response to both of the following questionnaire items: (1) “Have you ever been diagnosed with allergic conjunctivitis or hay fever?” and (2) either “Do you currently have conjunctivitis symptoms (itchy eyes, redness, excessive tearing, etc.)?” or “Are these symptoms particularly severe from February to April?”

**JCP** was defined as a positive response to either of the following sets of questionnaire items: (1) both (a) “Have you been diagnosed with hay fever?” and (b) “Are your hay fever symptoms (nasal, ocular, or systemic symptoms) particularly severe from February to April?”; or (2) both (a) “Have you been diagnosed with allergic rhinitis or rhinitis caused by hay fever?” or “Have you been diagnosed with conjunctivitis caused by allergic conjunctivitis or pollinosis?” and (b) “Are your nasal or ocular symptoms particularly severe from February to April?”

**FA** was defined as a positive response to the following questionnaire item: “Do you currently have a food allergy to the same food(s) previously diagnosed?”

**An** was defined using the same criterion, based on a positive response to the corresponding questionnaire item.

**Current allergic disease** was defined as the presence of 1 or more of the following conditions at the time of survey: BA, AD, AR, AC, or JCP.

“Major allergy” refers to a composite family history variable defined as the presence of bronchial asthma, atopic dermatitis, allergic rhinitis, or hives in any first-degree family member (father, mother, or siblings). This definition has been consistently used across all WJSAAC surveys to maintain methodological continuity. The variable was coded as positive if any of these conditions were reported in a first-degree family member and negative otherwise.

The questionnaires were distributed to each school and distributed to each child through their homeroom teacher. The children took them home, and parents filled out the questionnaires with questions about their children. The questionnaires were then collected by the homeroom teacher. During this period, contact with the schools was handled by a local pediatrician who was in charge of the survey.

Population density was categorized into 3 groups: high (≥1500 persons/km^2^), middle (250–1499 persons/km^2^), and low (<250 persons/km^2^).^7^ Statistical analysis was performed using SPSS 20.0J for Windows (SPSS Japan, Tokyo, Japan). Group differences in prevalence were initially assessed descriptively using the Chi-square test. To evaluate associations between family history of allergic diseases and the prevalence of allergic diseases, logistic regression analyses were performed. Crude odds ratios (cORs) and adjusted odds ratios (aORs) with 95% confidence intervals (CIs) and p-values were calculated. The adjusted models included sex, school grade (1–6), population density (low, middle, high), and respiratory infections before the age of 2 years as covariates. The results of these analyses are presented in Supplementary Tables 4-1 to 4-5.

## Results

Using the methods described above, we were able to obtain subjects who agreed to cooperate and participate in the survey from 55,388 to 29,553 elementary school students aged 6–12 years old from 11 prefectures in western Japan, and the response rate of the questionnaires was over 93%. Table 1 shows the number of subjects, gender, return rate, and a breakdown by region (population density).

**Table 1:** Characteristics of the subjects.

Survey Year	No. of school† n	No of children (people) (Age: 6–12 years)			Response rate (%)	Population density (people/km^2^)		
		Male n (%)	Female n (%)	Total n		≥1500 n (%)	250–1500 n (%)	<250 n (%)
1982 (WJSAAC-I)	70	28,036 (50.6)	27,352 (49.4)	55,388	95.9	20,421 (36.9)	31,545 (56.9)	3422 (6.2)
1992 (WJSAAC-II)	79	23,574 (50.5)	23,144 (49.5)	46,718	96.8	14,361 (30.7)	30,224 (64.7)	2113 (4.5)
2002 (WJSAAC-III)	81	18,264 (50.4)	17,964 (49.6)	36,228	96.1	11,994 (33.1)	22,706 (62.7)	1528 (4.2)
2012 (WJSAAC-IV)	81	17,217 (50.8)	16,685 (49.2)	33,902	96.2	9960 (29.4)	22,394 (66.1)	1548 (4.6)
2022 (WJSAAC-V)	75	15,079 (51.0)	14,474 (49.0)	29,553	92.8	9608 (32.5)	18,535 (62.7)	1410 (4.8)

WJSAAC: West Japan Study of Asthma and Allergies in Childhood.

^†^

† Changes in the number of schools were due to school reorganization over time, including branching and consolidation. In addition, 5 schools did not participate in the 2022 survey. The number of subjects, gender ratio, response rate, and breakdown by region (population density) are shown. The subjects were 55,388 to 29,553 elementary school students aged 6 to 12 years old in 11 prefectures in western Japan, and the response rate of the questionnaire exceeded 93%.

The prevalence of allergic diseases by gender and the overall prevalence of subjects in Phases I to V are shown in Table 2.

**Table 2:** Prevalence of allergic diseases.

Allergic Diseases	Prevalence (%)									
	male					female				
	1982	1992	2002	2012	2022	1982	1992	2002	2012	2022
Bronchial asthma	3.83	5.62	8.10	5.95	3.22	2.49	3.57	4.95	3.46	2.12
Atopic dermatitis		16.49	13.73	12.06	12.89		18.07	13.89	11.38	13.09
Allergic rhinitis		19.22	24.29	32.85	38.48		12.49	16.54	23.10	29.13
Allergic conjunctivitis		7.73	10.78	12.44	13.38		5.71	8.74	10.32	10.88
Japanese cedar pollinosis		4.25	6.36	10.62	15.80		3.00	5.09	9.18	12.95
Current allergic disease^a^		33.78	37.53	43.11	47.33		28.75	30.54	33.83	39.24

The prevalence of allergic diseases by gender and total among subjects in phases I to V is shown. In 1992, atopic dermatitis (AD) had the highest prevalence, followed by allergic rhinitis (AR), allergic conjunctivitis (AC), bronchial asthma (BA), and Japanese cedar pollinosis (JCP). By 2022, the order had changed, with AR showing the highest prevalence, followed by JCP, AD, AC, and BA. The prevalence of current allergic disease, defined as the presence of 1 or more of BA, AD, AR, AC, or JCP, increased from 31.27% in 1992 to 43.37% in 2022.

a Current Allergic Disease refers to having one or more of the following: BA, AD, AR, AC, or JCP

In 1992, the prevalence of currently symptomatic individuals (ie, current symptoms) was highest in male in the order AR, AD, AC, BA, and JCP, but by 2022, AR and JCP had increased, with the order being AR, JCP, AC, AD, and BA.

**Fig. 1:**
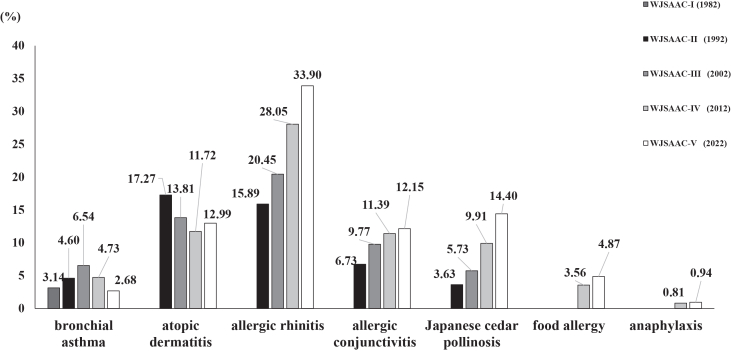
Trends in the prevalence of each allergic disease. The highest prevalence rates were allergic rhinitis (AR), Japanese cedar pollinosis (JCP), atopic dermatitis (AD), allergic conjunctivitis (AC), and bronchial asthma (BA) in 2022

For female, the order was AD, AR, AC, BA, and JCP in 1992, but in 2022, it is AR, AD, JCP, AC, and BA. Overall, the order was the same as for female in 1992, but by 2022 it was AR, JCP, AD, AC, and BA (Fig. 1).

**Fig. 2:**
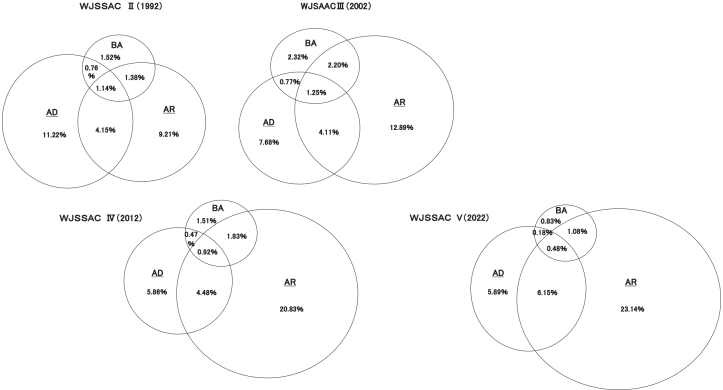
Prevalence rate of 3 allergic disease in WJSAACII ∼ V. BA: bronchial asthma, AD: atopic dermatitis, AR: allergic rhinitis, WJSAAC: West Japan Study of Asthma and Allergies in Childhood

The comorbidity rate of each allergic diseases with other allergic diseases were shown in Table 3. The combined prevalence of AR, AD, and BA rose from 29.4% in 1992 to 37.8% in 2022 (Fig. 2).

**Table 3:** Comorbidity Rate of each allergic diseases with other allergic diseases

Allergic disease	Bronchial Asthma (%)				Atopic Dermatitis (%)				Allergic Rhinitis (%)			
	1992	2002	2012	2022	1992	2002	2012	2022	1992	2002	2012	2022
Comorbidity												
Bronchial asthma					10.52	14.63	11.82	5.11	15.28	16.87	9.79	4.78
Atopic dermatitis	39.49	30.90	29.32	24.75					33.31	26.20	19.23	19.91
Allergic rhinitis	52.74	52.76	58.08	60.48	30.64	38.80	46.01	51.98				
Allergic conjunctivitis	23.67	24.40	24.77	26.64	14.61	19.53	21.21	21.76	24.56	30.60	30.02	28.67
Japanese cedar pollinosis	11.91	12.45	17.22	21.21	7.71	11.31	16.81	22.75	19.97	24.61	32.75	39.82

Allergic disease	Allergic Conjunctivitis (%)				Japanese Ceder Pollinosis (%)			
	1992	2002	2012	2022	1992	2002	2012	2022
Comorbidity								
Bronchial asthma	16.19	16.34	10.28	5.87	15.09	14.21	8.22	3.95
Atopic dermatitis	37.51	27.61	21.82	23.25	36.67	27.26	19.89	20.51
Allergic rhinitis	58.00	64.08	73.91	79.96	87.38	87.81	92.74	93.73
Allergic conjunctivitis					73.23	72.21	61.24	52.55
Japanese cedar pollinosis	39.52	42.37	53.25	62.28

**Fig. 3:**
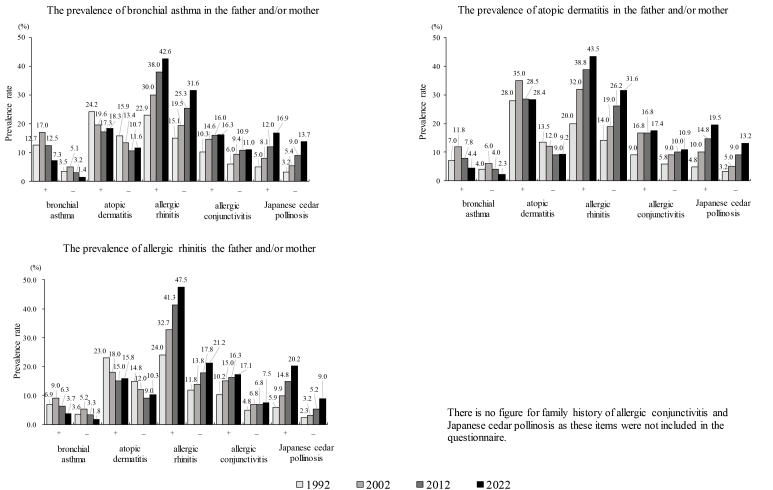
Prevalence of allergic diseases by family history of allergic diseases (either or both parents). In the group with a family history of allergic diseases, the prevalence was consistently higher throughout all survey phases. For example, bronchial asthma (BA) was more strongly associated with those with a history of bronchial asthma compared with those with a family history of other allergies. There is no figure for family history of allergic conjunctivitis and pollinosis as these items were not included in the questionnaire.

**Fig. 4:**
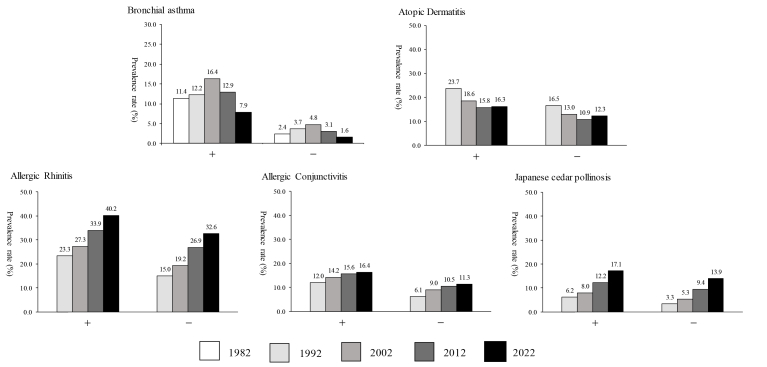
Prevalence rate of allergic diseases by frequent respiratory infections under the age of 2 years. In the infection-positive group, the prevalence rate of other allergic diseases was also significantly higher

The male-to-female ratio in the prevalence for each allergic disease, except AD in 1992, 2002 and 2022, indicated a higher prevalence in males (Table 4). Changes in prevalences were observed in AR, AC, and JCP, with a higher prevalence from Phase II to Phase IV as the school grades advanced, but such changes was not observed for other allergic diseases (Supplementary Fig. 1). The regional differences in prevalence rates have diminished (Supplementary Table 2 and 3). The prevalences of allergic diseases in groups with and without a family history of allergic diseases are shown in Fig. 3. The prevalence was significantly higher in groups with a family history of allergic diseases, which was observed consistently across all survey phases. For example, BA was more strongly associated with those having a family history of BA compared with those with a family history of other allergies (Supplementary Table 4-1 to 4-5). Changes in the relationship between heating, air conditioning, nutrition, infections, smoking, pets, carpets, and prevalence rates from Phase I to Phase V are shown in Table 5. The prevalence of allergic diseases according to early childhood infections, defined as a history of respiratory infections before the age of 2 years, is illustrated in Fig. 4. Bronchial asthma showed the largest difference in prevalence between children with and without early childhood respiratory infections, and higher prevalence was also observed for other allergic diseases in the infection-positive group.

**Table 4:** Gender ratio in the prevalence for each allergic disease

Survey Year	Bronchial Asthma (%)			Atopic Dermatitis (%)			Allergic Rhinitis (%)			Allergic Conjunctivitis (%)			Japanese Cedar Pollinosis (%)		
	male	female	m/f	male	female	m/f	male	female	m/f	male	female	m/f	male	female	m/f
**1992**	**5.62**	**3.57**	**1.57**	**16.49**	**18.07**	**0.91**	**19.22**	**12.49**	**1.54**	**7.73**	**5.71**	**1.35**	**4.25**	**3.00**	**1.42**
**2002**	**8.1**	**4.95**	**1.64**	**13.73**	**13.89**	**0.99**	**24.29**	**16.54**	**1.47**	**10.78**	**8.74**	**1.23**	**6.36**	**5.09**	**1.25**
**2012**	**5.95**	**3.46**	**1.72**	**12.06**	**11.38**	**1.06**	**32.85**	**23.10**	**1.42**	**12.44**	**10.32**	**1.21**	**10.62**	**9.18**	**1.16**
**2022**	**3.22**	**2.12**	**1.52**	**12.89**	**13.09**	**0.98**	**38.48**	**29.13**	**1.32**	**13.38**	**10.88**	**1.23**	**15.80**	**12.95**	**1.22**

**Table 5:** Prevalence rate of each allergic disease by environment from phase II to phase V

	Survey year	Bronchial asthma, n (%)					Atopic dermatitis, n (%)				Allergic rhinitis, n (%)				Allergic conjunctivitis, n (%)				Japanese ceder pollinosis, n (%)				Current allergic disease, n (%)				Cumulative allergic disease, n (%)			
		1982	1992	2002	2012	2022	1992	2002	2012	2022	1992	2002	2012	2022	1992	2002	2012	2022	1992	2002	2012	2022	1992	2002	2012	2022	1992	2002	2012	2022
Heating	Clean type	3.10	4.40	6.19	4.70	2.70	16.52	13.46	11.68	13.09	14.71	19.15	28	33.7	6.29	8.92	11.47	12.12	3.15	5.19	10.02	14.25	29.68	32.56	38.51	43.32	43.66	47.41	50.55	54.3
	Mixed type	3.27	4.76	6.98	4.56	2.70	18.84	14.06	12.45	11.76	16.39	22.29	30.1	36.41	6.71	11.08	12.34	13.53	3.51	6.58	10.98	16.4	33.02	35.93	40.94	44.53	47.85	51.36	53.44	54.92
	Dirty type	3.14	5.22	6.83	5.28	2.44	17.93	14.52	12.44	12.75	16.87	21.22	27.07	33.37	7.54	10.04	10.86	11.4	4.23	5.87	8.82	15.06	32.86	35.50	38.01	42.68	47.75	49.48	49.9	53.45
Air conditioning	None		4.39	6.22	5.64	2.60	15.81	11.87	11.10	12.64	14.20	18.08	23.65	31.85	6.47	7.77	8.22	11.11	3.36	4.55	6.54	13.33	28.91	29.98	34.39	38.89	42.09	43.56	45.74	48.15
	Electric air conditioning		4.81	6.52	4.71	2.68	18.35	13.89	11.73	12.86	16.34	20.81	28.38	33.85	6.91	10.02	11.67	12.19	3.51	5.82	10.17	14.45	32.62	34.44	38.86	43.32	47.48	48.97	50.81	54.25
	Central cooling		4.55	7.53	2.74	0.00	15.88	18.28	8.90	17.05	16.21	18.28	25.34	37.5	6.29	6.45	7.53	13.64	4.55	5.38	8.9	14.77	30.11	32.26	35.62	47.73	44.91	48.92	52.05	56.82
	Other		3.54	6.84	4.52	3.59	16.38	14.53	12.50	16.03	13.33	20.25	27.88	36.56	5.22	9.82	10.95	13.55	2.72	6.12	9.67	15.31	27.99	35.13	38.54	46.81	42.46	49.57	51.9	57.6
Nutrition	Breast feeding		4.88	6.99	4.62	2.76	18.33	14.58	12.74	13.61	15.79	21.29	28.25	34.71	6.89	10.30	11.64	12.78	3.65	6.02	10.35	15.19	32.26	35.44	39.5	44.47	46.75	49.90	51.83	55.31
	Mixed feeding		4.52	5.88	4.85	2.54	16.84	13.24	11.11	12.33	16.29	18.67	28.31	33.19	6.79	8.50	11.34	11.55	3.71	4.86	9.4	13.72	31.16	32.05	38.36	42.44	45.88	45.98	50.25	53.53
	Formula feeding		4.44	6.36	5.12	2.93	16.60	13.33	10.16	12.58	16.13	20.49	27.86	33.35	6.66	9.79	11.29	11.58	3.62	5.82	10.46	13.48	30.83	33.71	37.14	42.18	43.73	48.39	49.29	52.79
Respiratory infections before age 2	+	11.42	12.23	16.35	12.91	7.89	23.68	18.62	15.85	16.31	23.33	27.33	33.93	40.15	11.95	14.21	15.62	16.43	6.17	7.96	12.2	17.14	43.88	45.85	48.34	51.76	59.88	62.06	62.41	65.2
	–	2.40	3.67	4.77	3.05	1.61	16.54	12.97	10.87	12.29	14.99	19.23	26.85	32.67	6.11	8.98	10.53	11.36	3.33	5.34	9.45	13.92	29.79	31.98	36.56	41.7	43.81	46.12	48.23	52.1
Asthmatic bronchitis	+	11.77	34.23	35.84	24.64	17.89	33.65	25.05	21.72	22.15	40.99	42.24	46.94	51.22	18.14	19.80	19.26	20.91	9.16	10.88	14.69	20.17	69.91	68.37	66.13	67.36	83.41	81.02	79.87	82.24
	–	1.38	0.87	1.44	0.89	0.37	15.21	11.85	9.80	11.44	12.73	16.66	24.41	30.85	5.29	8.02	9.88	10.85	2.94	4.83	8.99	13.41	26.41	28.10	33.23	39.29	70.70	40.27	44.96	49.75
Family smoking	+	3.24	4.58	6.49	4.91	2.85	17.27	14.32	10.97	12.64	15.25	21.99	25.6	32.39	6.21	11.18	10.28	11.58	3.40	6.60	8.65	13.27	30.71	35.89	36.13	41.61	45.02	47.05	47.71	51.95
	–	3.05	4.62	6.58	4.64	2.63	17.27	13.44	12.09	13.07	16.68	19.17	29.26	34.4	7.37	8.58	11.95	12.41	3.91	4.99	10.53	14.81	31.98	32.64	39.74	43.95	46.03	50.19	52.03	55.05
Pets	+			5.87	4.73	2.84		13.14	10.69	12.15		19.62	27.86	32.96		9.40	11.51	11.63		5.45	9.81	13.67		32.53	37.74	41.58		47.49	49.57	52.39
	–			6.97	4.73	2.63		14.25	12.13	13.28		21.00	28.13	34.27		10.01	11.35	12.42		5.91	9.95	14.72		35.07	38.86	44.04		48.63	51.01	55.01
Cat	Indoor			5.72	5.24	2.47		11.25	9.74	10.44		18.03	25.38	30.98		7.37	8.75	10.5		4.87	7.82	11.71		28.95	34.66	37.9		43.36	47.25	49.66
	Outdoor			6.32	2.73	1.67		13.90	11.26	16.67		17.85	22.87	37.7		8.53	9.22	12.3		4.90	5.46	13.11		30.81	34.47	50		44.87	44.71	58.2
	None			6.58	4.72	2.70		13.92	11.82	13.16		20.61	28.22	34.14		9.90	11.54	12.34		5.78	10.05	14.66		34.36	38.77	43.79		48.46	50.82	54.67
Carpet	+			5.16	4.91	2.67		13.22	11.40	11.81		18.67	26.89	34.01		8.56	10.78	12.22		5.61	9.49	14.69		31.47	37.36	42.93		45.53	49	53.72
	–			7.04	4.67	2.70		14.02	11.88	13.52		21.15	28.56	33.87		10.19	11.68	12.21		5.77	10.09	14.33		35.03	39.12	43.63		49.25	51.36	54.62

The following describes changes in the prevalence of individual allergic diseases.

## Prevalence of AD

The prevalence of AD decreased for both genders from 1992 onwards (Table 2), with slight decreases observed between grades 1 and 6. The male-to-female ratio remained approximately 1.0 across Phase II to Phase V. Okinawa consistently had the lowest prevalence across all phases. The prevalence of AD showed no differences based on population density, smoking status, heating, or air conditioning. A higher prevalence of AD was observed among children with a family history of allergic diseases. In particular, a family history of eczema showed a strong association with AD prevalence. A higher prevalence was also observed among children with major allergies (BA, AR, and AD) in their family history (Supplementary Tables 4-1 to 4-5). The male-to-female ratio in the prevalence has increased from 0.91 in 1992 to 0.98 in 2022 (Table 4). Higher prevalence was observed in breastfeeding groups compared with formula-fed groups across 3 surveys. A higher prevalence was also observed in groups with a history of respiratory diseases (Table 5).

## Prevalence of AR

In 2022, the prevalence of AR was 38.5% in boys (19.2% in 1992) and 29.2% in girls (12.5% in 1992), with an overall prevalence of 33.9%. The prevalence in first graders was 12.2%, with an increased rate (prevalence in sixth graders divided by the prevalence in first graders times 100) of 50.4. The male-to-female ratio was 1.5. All prefectures showed an increase in prevalence, with Okinawa consistently having the lowest prevalence (Supplementary Table 2). There were no significant results related to smoking, air conditioning, heating, or nutrition (Table 5). AR was associated with a family history of AR. Children with a family history of other allergic diseases also showed a higher prevalence of AR (Supplementary Tables 4-1 to 4-5). In groups with a family history of allergies, the prevalence was 34.4% in non-smoking groups. A higher prevalence was also observed in groups with a history of respiratory diseases.

## Prevalence of AC

The prevalence of AC increased for both genders and overall, across Phase II to Phase V, with an increased rate of 45.0%–58.8% and a male-to-female ratio of 1.2:1.4. No differences were observed based on smoking, heating, or infant nutrition, but non-smoking and air-conditioned groups had a slightly higher prevalence (Table 5).

## Prevalence of JCP

The prevalence of JCP increased for both genders and overall, across Phase II to Phase V, with an increased rate of 66.7%–100% from first to sixth grade and a male-to-female ratio of 1.0:1.4. Okinawa consistently had the lowest prevalence across all phases, whereas Hyogo and Kagawa had a higher prevalence rate (Supplementary Table 3).

## Prevalence of FA and An

In WJSAAC-IV (2012) and WJSAAC-V (2022), the prevalence of food allergy (FA) was 3.6% and 4.9%, respectively, indicating an increasing trend over the past decade. The prevalence of anaphylaxis (An) showed a slight increase from 0.8% to 0.9%.

## Discussion

It has been long recognized that the prevalence of allergic diseases is increasing worldwide. Despite advances in understanding the pathophysiology of these diseases and improvements in various treatments, the global trend of increasing allergic diseases continues to be reported, although BA has begun to show a declining trend.

The data and analysis of Phase I to V of BA have been reported in detail^7^ in the World Allergy Organization Journal, so we will summarize the main points here.

In western Japan, the prevalence of BA increased from 3.2% in 1982 to 4.6% in 1992 and 6.5% in 2002, based on the Japanese version of the ATS-DLD questionnaire, which, although yielding lower prevalence estimates than ISAAC, enables the identification of definite BA cases.^8^^,^^9^ BA began to decrease from Phase IV onwards. In Phase V, reductions in all prefectures, genders, and grades were observed, even in Okinawa, which had not shown a decrease in Phase IV. ISAAC Phase III trials also indicated a declining trend in regions with a high prevalence in Phase I.^10^ More recently, the Global Asthma Network (GAN), which continued the ISAAC methodology, reported regional heterogeneity and stabilization or decline of asthma prevalence in several high-income countries.^11^ The strict definition of BA in ATS-DLD surveys might have missed milder BA cases, but the prevalence of W showed similar trends to BA.^12^ This pattern is consistent with ISAAC and GAN findings of stabilization or decline in high-income regions.

Overall, BA prevalence increased from Phase I to Phase III and declined from Phase IV onward. Male predominance was consistently observed, and BA was more frequent in children with early childhood respiratory infections and a family history of allergic diseases. Notably, the age of onset shifted toward younger ages, with the proportion of children developing symptoms at ≤2 years increasing from 15.0% in 1982 to 37.2% in 2022.^13^

On the other hand, for other allergic diseases, the disease definitions and methods for epidemiological surveys using questionnaires have not been fully established, and there is a wide variation in the reports to date.

In the ISAAC survey, AD is referred to as atopic eczema, defined as “an itchy rash that has come and gone over the past 12 months.” Our ISAAC survey in 1995 found that Japan had a high prevalence compared with other countries.^3^ In the western Japan epidemiological survey, AD was defined as “having been diagnosed with eczema or AD by a doctor and currently having it,” with prevalence rates of 17.3% in 1992, 13.8% in 2002, 11.8% in 2012, and 13.0% in 2022. When the same population was assessed using the ISAAC method, the prevalence rate for AD was 16%, showing no significant difference compared to the results of the current survey method.^6^ In our recent survey, the prevalence of AD ranged from a minimum of 7.4% in Okinawa to a maximum of 16.0% in Hyogo. The low prevalence in Okinawa for AR, AC, and JCP can be explained by the absence of cedar and cypress pollen, but the low AD prevalence, half that of other prefectures in previous surveys, remains an intriguing topic for future analysis. The persistently low prevalence in Okinawa warrants further investigation of regional environmental and cultural factors.

Our survey showed a decrease in AD prevalence since 2002. Domestically, Kurosaka et al reported a clear decrease in AD prevalence among first-grade students in Himeji City over time,^14^ and a large-scale survey of elementary school children in Osaka by Yura et al reported no increase in AD prevalence since the 1990s, with no differences by grade.^15^ To analyze the reasons for the continued decrease in AD prevalence, it is essentials to examine its relationship with FA in early childhood and factors such as recent detailed skin care guidance and widespread adoption, the advent of tacrolimus ointments, reduced steroid phobia, and the widespread adoption of treatment guidelines leading to the early mitigation of AD symptoms by school age.

In the ISAAC survey, AR was investigated together with AC as allergic rhino-conjunctivitis. The definition of AR was “having trouble with sneezing, a runny nose, or nasal congestion in the past 12 months apart from when you had a cold,” with prevalence rates of 25.6% for ages 6–7 years and 41.0% for ages 13–14 years in Japan. For those with eye symptoms (itchy eyes or watery eyes), the prevalence was 7.9% for ages 6–7 years and 15.6% for ages 13–14 years.^9^ The definition of AR in the western Japan epidemiological survey is described in the methods section, and the prevalences of AR, AC, and JCP have been increasing continuously. In regions with high cedar pollen dispersal, sensitization rates have increased over time,^16^ accompanied by higher prevalence of AR, AC, and JCP outside Okinawa. Although JCP would theoretically be rare in Okinawa due to the absence of cedar pollen dispersal, a prevalence of over 1% was observed. This may reflect migration from pollen-dispersal regions. The increase may reflect both perennial (mite-related) and seasonal (pollen-related) components, and further sero-epidemiological studies measuring specific sensitization are warranted.

Considering the rate of increase or decrease in allergic disease prevalence using Phase II as a baseline (1.0), BA was decreased by 0.6 times, AD by 0.8 times, and AR increased by 2.1 times, AC by 1.8 times, and JCP by 4.0 times. Overall allergic diseases prevalence were increased by 1.4 times, indicating that the increases in AR, AC, and JCP were largely related to increased allergens, particularly cedar and cypress pollen.^17^ National monitoring data have demonstrated substantial increases in pollen dispersal over time.^18^ No measure has been shown to reduce cedar and cypress pollen in Japan; therefore, AR and AC caused by pollen are likely to continue increasing.

FA and An have also been attracting attention recently.^19^ These conditions were newly included in Phases IV and V. Although FA showed an increasing trend over the past decade, detailed analyses will be reported separately, as the present study focuses on long-term trends in major allergic diseases.

When examining the prevalence of allergic diseases and major confounding factors, the previously observed correlation between air pollution (such as suspended particulate matter (SPM), sulfur oxide (SOx), and nitrogen oxide (NOx)) and BA was analyzed. A correlation with NOx and SPM was observed in 1992 but disappeared after 2002. Pollutant levels declined over time in the surveyed areas, and no clear trend was observed in vehicle numbers, suggesting that additional environmental factors or qualitative changes in pollutants should be considered in future analyses.

Considering trends in prevalence of allergic diseases in children from first to sixth school grade, as shown in Supplementary Fig. 1, BA remained unchanged, AD decreased, and AR, AC, and JCP clearly increased with advancing grade. This suggests that AR, AC, and JCP will continue to increase; however, the reasons for the decrease in BA and AD and their future trends should be examined. Comparison of ISAAC Phases I and III showed that BA increased in low-prevalence regions among 6–7 years age group, together with rising trends in AR, AC, and AD. In contrast, among 13–14 years age group, BA declined in previously high-prevalence regions, while AR and AC remained largely unchanged and AD showed mixed trends,^10^ The impact of airway infections up to the age of 2 years on BA, AR, and AD is an important point. Controlling infections in this age group may be effective at preventing the development of allergic diseases^18^^,^^20^. As suggested by the hygiene hypothesis, infections are not uniformly detrimental, and their timing and type may influence allergy prevalence. Further detailed analyses of respiratory tract infections are warranted.

The decreasing trends in BA and AD prevalence in regions with strong hygiene orientation in Japan might indicate the weaknesses of the hygiene hypothesis.^21^ AR, AC, and JCP may be more closely related to Th2-mediated mechanisms, whereas BA and AD may be influenced by infections and environmental factors. These differing trends highlight the need for further analysis. Similarly, the possibility that pet ownership might suppress the onset of allergies has been suggested.^22^ The 2002 data showed a higher allergy prevalence in non-pet-owning groups, and in 2012, only BA prevalence was higher in indoor pet-owning groups. Whether this supports the hygiene hypothesis or reflects the efforts of affected individuals to maintain their environment by removing allergens remains unclear. The difference was not sufficiently significant to provide uniform clinical guidance.

Family history remains an important risk factor, showing organ specificity and disease specificity. Individuals with a family history of BA have a higher risk of BA compared with those without it, even when compared with those with a family history of other allergic diseases, consistently across 3 surveys.

Even considering recall bias, the ORs are high, suggesting the genetic transmission of factors determining organ hypersensitivity and the importance of organ-specific treatment strategies.

Regarding regional differences, Okinawa's prevalence rates were markedly lower, whereas Hyogo and Kagawa had higher rates for AC and JCP. However, differences observed by population density in 1992 had disappeared by 2002, 2012, and 2022. Factors such as living environment, diet, indoor and outdoor activity time, and air quality require analysis. It appears that the standardization of living and dietary environments in Japan has reduced these differences, but objective indicators are insufficient for interpretation.

## Limitations of this study

The diagnosis in this study was based solely on a questionnaire, and the limitations of epidemiological surveys using questionnaires, which are based on the respondent's subjective opinion, are unavoidable. In addition, no actual clinical or laboratory diagnosis was performed. Recently, the ISAAC questionnaire has been used in many cases. This study was conducted before that, so the ATS-DLD questionnaire, which was often used at the time, was used. In Japan, it is still used as a highly reliable questionnaire.

In addition, this study was conducted on a large number of people in 11 prefectures in western Japan, and the data from Okinawa Prefecture was different from the others. We would like to clarify these points by conducting a more detailed analysis in the future, rather than simply describing the environmental and geographical characteristics.

The strengths of this study, although it has the limitations mentioned above, are that it is a regular survey of a large number of people, over 40 years, using the same method, the same target area, and the majority of the people in charge were pediatricians involved in allergy treatment in the area, and the response rates were all over 90%, which is a major strength. Such reports are not within the scope of the authors' investigation, and are considered to be valuable.

The potential impact of the COVID-19 pandemic, including reduced respiratory infections and changes in lifestyle and social behavior, should be considered when interpreting Phase V findings, as lifestyle modifications during the pandemic may have influenced allergic disease prevalence.^7^ The continuation of epidemiological surveys using the same regions and methodologies, and active participation in international epidemiological surveys, such as ISAAC for global comparisons, are expected to elucidate various factors and contribute to treatment strategies.

## Availability of data and materials

The datasets used and/or analyzed during the current study are available from the corresponding author on reasonable request.

## Authors' contributions

SN, HO, HM and JT wrote the manuscript. SN, HO, HM and JT designed the study. SN, HO, HM and JT collected data, and SN, HO, HM and JT performed statistical analyses and interpreted results. All authors read and approved the final manuscript.

## Ethics approval and consent to participate

This study adhered to the principles outlined in the Declaration of Helsinki and the ethical guidelines for medical and health research involving human participants established by the Ministry of Health, Labour and Welfare of Japan. This study was approved by the ethics committee of Fukuoka Children's Hospital (ID: 2021-963).

## Consent for publication

Not applicable.

## Declaration of generative AI and AI-assisted technologies in the writing process

Nothing to disclose.

## Funding

This work was supported by a science research grant for research on allergic diseases and immunology from the 10.13039/501100003478Ministry of Health, Labour, and Welfare in Japan (grant number: 20FE2001) and the 10.13039/100014423Environmental Restoration and Conservation Agency of Japan.

## Declaration of competing interests

The authors declare they have no conflicts of interest associated with this manuscript.
