# Supplementary figures and images for: Trends in the prevalence of childhood allergic diseases in Japan: Comparison of surveys conducted in 1982, 1992, 2002, 2012, and 2022 (WJSAAC phase I–V)
Source: World Allergy Organ J. 2026 May 14;19(6):101396. doi: 10.1016/j.waojou.2026.101396 (PMC13196322; doi:10.1016/j.waojou.2026.101396)

## Slide 1
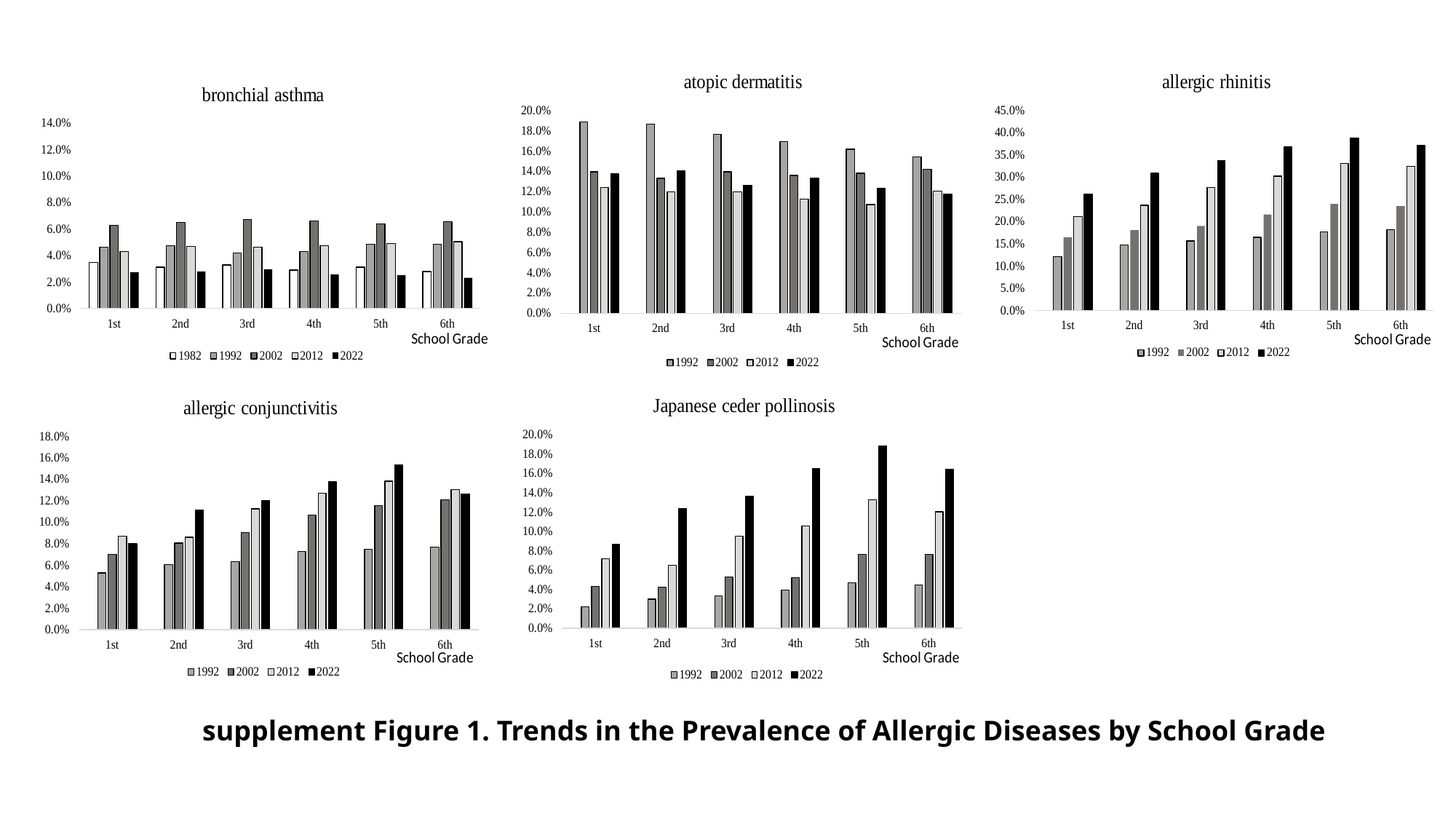

supplement Figure 1. Trends in the Prevalence of Allergic Diseases by School Grade

Supplement: Multimedia component 1 [file mmc1.pptx]
