# Supplementary material for: Trends in the prevalence of childhood allergic diseases in Japan: Comparison of surveys conducted in 1982, 1992, 2002, 2012, and 2022 (WJSAAC phase I–V)
Source: World Allergy Organ J. 2026 May 14;19(6):101396. doi: 10.1016/j.waojou.2026.101396 (PMC13196322; doi:10.1016/j.waojou.2026.101396)
Supplement: Multimedia component 2 [file mmc2.doc]

| supplement Table 1. The prevalence of each allergic disease when comorbid with other allergic diseases | | | | | | | | | | | | |
| --- | --- | --- | --- | --- | --- | --- | --- | --- | --- | --- | --- | --- |
|  | bronchial asthma (%) | | | | atopic dermatitis (%) | | | | allergic rhinitis  (%) | | | |
|  | 1992 | 2002 | 2012 | 2022 | 1992 | 2002 | 2012 | 2022 | 1992 | 2002 | 2012 | 2022 |
| Bronchial Asthma |  |  |  |  | 1.82 | 2.02 | 1.39 | 0.66 | 2.43 | 3.45 | 2.75 | 1.62 |
| Atopic Dermatitis | 1.82 | 2.02 | 1.39 | 0.66 |  |  |  |  | 5.29 | 5.36 | 5.39 | 6.75 |
| Allergic Rhinitis | 2.43 | 3.45 | 2.75 | 1.62 | 5.29 | 5.36 | 5.39 | 6.75 |  |  |  |  |
| Allergic Conjunctivitis | 1.09 | 1.60 | 1.17 | 0.71 | 2.52 | 2.70 | 2.49 | 2.83 | 3.90 | 6.26 | 8.42 | 9.72 |
| Japanese Cedar Pollinosis | 0.55 | 0.81 | 0.81 | 0.57 | 1.33 | 1.56 | 1.97 | 2.95 | 3.17 | 5.03 | 9.19 | 13.50 |
|  |  |  |  |  |  |  |  |  |  |  |  |  |

| supplement Table2. Prevalence of Allergic Diseases and Population Density (Phases I to V) | | | | | | | |
| --- | --- | --- | --- | --- | --- | --- | --- |
| population density (people/km^2^) | phase | n | bronchial asthma  (%) | atopic dermatitis (%) | allergic rhinitis (%) | allergic conjunctivitis (%) | Japanese cedar pollinosis (%) |
|  | Ⅰ | 20,420 | 3.9 | - | - | - | - |
|  | Ⅱ | 14,361 | 5.2 | 18.4 | 18.2 | 7.2 | 3.5 |
| ≥1,500 | Ⅲ | 11,994 | 6.5 | 14.8 | 20.9 | 9.2 | 4.9 |
|  | Ⅳ | 9,960 | 4.7 | 12.3 | 27.4 | 11.0 | 9.1 |
|  | Ⅴ | 9,608 | 2.4 | 13.6 | 34.3 | 12.2 | 13.5 |
|  | Ⅰ | 31,546 | 2.8 | - | - | - | - |
|  | Ⅱ | 30,224 | 4.4 | 16.9 | 15.0 | 6.6 | 3.8 |
| 250-1,500 | Ⅲ | 22,706 | 6.6 | 13.2 | 20.4 | 10.1 | 6.1 |
|  | Ⅳ | 22,394 | 4.6 | 11.4 | 28.5 | 11.5 | 10.3 |
|  | Ⅴ | 18,535 | 2.8 | 12.8 | 33.6 | 12.2 | 14.8 |
|  | Ⅰ | 3,422 | 1.9 | - | - | - | - |
|  | Ⅱ | 2,113 | 4.0 | 15.1 | 12.7 | 5.5 | 3.0 |
| <250 | Ⅲ | 1,528 | 6.3 | 15.4 | 18.2 | 10.0 | 6.0 |
|  | Ⅳ | 1,548 | 6.2 | 12.7 | 26.7 | 12.3 | 9.1 |
|  | Ⅴ | 1,410 | 3.8 | 11.3 | 35.6 | 11.6 | 15.7 |

Supplement Table3. Prevalence of Each Allergic Disease by Prefecture

| **prefecture** | **Bronchial Asthma  (%)** | | | | | **Atopic Dermatitis  (%)** | | | | **Allergic Rhinitis  (%)** | | | | **Allergic Conjunctivitis  (%)** | | | | **Japanese Cedar Pollinosis  (%)** | | | |
| --- | --- | --- | --- | --- | --- | --- | --- | --- | --- | --- | --- | --- | --- | --- | --- | --- | --- | --- | --- | --- | --- |
|  | **1982** | **1992** | **2002** | **2012** | **2022** | **1992** | **2002** | **2012** | **2022** | **1992** | **2002** | **2012** | **2022** | **1992** | **2002** | **2012** | **2022** | **1992** | **2002** | **2012** | **2022** |
| **Fukuoka** | **3.91** | **5.10** | **6.67** | **4.50** | **2.39** | **18.11** | **14.31** | **12.47** | **13.38** | **16.40** | **21.54** | **27.29** | **33.82** | **7.16** | **10.00** | **12.21** | **13.20** | **3.42** | **5.75** | **9.99** | **15.44** |
| **Saga** | **2.48** | **3.85** | **6.28** | **5.03** | **3.11** | **17.06** | **14.70** | **11.36** | **12.63** | **13.08** | **20.71** | **29.74** | **40.91** | **6.45** | **11.06** | **13.19** | **13.35** | **3.87** | **7.16** | **12.97** | **17.13** |
| **Nagasaki** | **3.72** | **5.20** | **6.69** | **4.39** | **2.63** | **20.07** | **15.03** | **12.34** | **13.88** | **19.27** | **20.67** | **24.34** | **30.94** | **6.85** | **8.99** | **9.76** | **10.63** | **3.90** | **5.36** | **8.75** | **14.08** |
| **Kumamoto** | **3.02** | **4.60** | **6.58** | **4.80** | **2.10** | **14.73** | **12.96** | **11.76** | **13.03** | **16.93** | **19.41** | **31.42** | **34.72** | **7.19** | **11.76** | **15.49** | **13.78** | **4.37** | **5.65** | **10.41** | **14.64** |
| **Kagoshima** | **3.20** | **4.58** | **6.82** | **4.43** | **2.82** | **15.51** | **13.20** | **11.19** | **14.08** | **12.95** | **19.16** | **29.73** | **34.13** | **7.68** | **8.73** | **7.12** | **8.23** | **2.92** | **4.15** | **4.79** | **7.30** |
| **Oita** | **2.66** | **4.97** | **6.73** | **4.88** | **3.26** | **18.74** | **13.00** | **13.89** | **12.63** | **17.39** | **22.27** | **34.47** | **37.08** | **4.71** | **7.02** | **12.97** | **11.72** | **3.75** | **6.14** | **14.59** | **18.11** |
| **Miyazaki** | **2.97** | **4.33** | **7.65** | **4.82** | **2.62** | **15.61** | **10.75** | **10.20** | **11.29** | **17.08** | **23.50** | **29.96** | **33.60** | **6.20** | **9.93** | **10.95** | **11.74** | **3.63** | **6.83** | **10.39** | **14.91** |
| **Yamaguchi** | **2.38** | **4.22** | **8.00** | **5.02** | **2.59** | **17.56** | **13.44** | **10.56** | **11.59** | **13.99** | **18.33** | **24.03** | **27.74** | **4.89** | **8.04** | **8.33** | **10.92** | **2.90** | **3.91** | **7.25** | **11.13** |
| **Okinawa** | **2.58** | **3.45** | **4.76** | **6.29** | **3.04** | **9.36** | **6.94** | **7.05** | **7.35** | **12.03** | **13.05** | **15.99** | **24.52** | **5.27** | **4.88** | **5.53** | **6.64** | **0.99** | **1.00** | **1.08** | **2.26** |
| **Hyogo** | **2.26** | **3.80** | **5.92** | **5.36** | **3.62** | **17.89** | **14.92** | **15.15** | **15.98** | **14.51** | **21.58** | **37.80** | **36.24** | **8.87** | **15.29** | **18.36** | **16.14** | **4.08** | **10.23** | **19.71** | **23.56** |
| **Kagawa** | **2.97** | **4.36** | **4.60** | **3.84** | **2.64** | **20.16** | **17.17** | **11.83** | **15.08** | **18.64** | **24.58** | **32.20** | **39.94** | **7.84** | **14.00** | **13.58** | **19.22** | **6.03** | **10.07** | **14.13** | **22.16** |
| **total** | **3.14** | **4.60** | **6.54** | **4.72** | **2.68** | **17.27** | **13.81** | **11.76** | **12.99** | **15.89** | **20.45** | **28.06** | **33.90** | **6.73** | **9.77** | **11.40** | **12.15** | **3.63** | **5.73** | **9.90** | **14.40** |

|  | | | | | | | | | | | | | | | | | | | | | | | | | | | | | | | | | | | | | | | |  | | | | | | | | | | | | |
| --- | --- | --- | --- | --- | --- | --- | --- | --- | --- | --- | --- | --- | --- | --- | --- | --- | --- | --- | --- | --- | --- | --- | --- | --- | --- | --- | --- | --- | --- | --- | --- | --- | --- | --- | --- | --- | --- | --- | --- | --- | --- | --- | --- | --- | --- | --- | --- | --- | --- | --- | --- | --- |
|  | |  | | | | | | | | | | | | | | | | | | |  | |  | | | |  | | | | | |  | | | | | | |  | | | | | | | | | | | | |
| supplement Table 4-1. Family History of Allergies and Prevalence of Bronchial Asthma (1982) | | | | | | | | | | | | | | | | | | | | | | | | | | | | | | | | | | |  | | | | | |  | | | | |  | | |  | | | |
| Family History | | | | | | | | | | | | | | | | | n | | | odds ratio | | | | | | Bronchial Asthma | | | | | | | |  | |  | | | | |  | | | | |  | | |  | | | |
| Major Allergy^†^ | | | | | | | | + | | | | | | | | | 23,069 | | | cOR (95%CI) | | | | | | 2.60 (2.35-2.88) | | | | | | | |  | |  | | | | |  | | | | |  | | |  | | | |
|  |  |  |  |  |  |  |  | - | | | | | | | | | 32,319 | | | aOR (95%CI) | | | | | | 2.31 (2.08-2.56) | | | | | | | |  | |  | | | | |  | | | | |  | | |  | | | |
| Bronchial Asthma | | | | | | | | + | | | | | | | | | 5,952 | | | cOR (95%CI) | | | | | | 3.98 (3.58-4.43) | | | | | | | |  | |  | | | | |  | | | | |  | | |  | | | |
|  |  |  |  |  |  |  |  | - | | | | | | | | | 49,436 | | | aOR (95%CI) | | | | | | 3.55 (3.19-3.96) | | | | | | | |  | |  | | | | |  | | | | |  | | |  | | | |
| Atopic Dermatitis | | | | | | | | + | | | | | | | | | 6,453 | | | cOR (95%CI) | | | | | | 1.90 (1.68-2.14) | | | | | | | |  | |  | | | | |  | | | | |  | | |  | | | |
|  |  |  |  |  |  |  |  | - | | | | | | | | | 48,935 | | | aOR (95%CI) | | | | | | 1.64 (1.45-1.86) | | | | | | | |  | |  | | | | |  | | | | |  | | |  | | | |
| Allergic Rhinitis | | | | | | | | + | | | | | | | | | 9,108 | | | cOR (95%CI) | | | | | | 2.35 (2.11-2.60) | | | | | | | |  | |  | | | | |  | | | | |  | | |  | | | |
|  |  |  |  |  |  |  |  | - | | | | | | | | | 46,280 | | | aOR (95%CI) | | | | | | 2.10 (1.89-2.34) | | | | | | | |  | |  | | | | |  | | | | |  | | |  | | | |
| Phase I n = 55,388 | | | | | | | |  | | | | | | | | |  | | |  | | | | | |  | | | | | | | |  | |  | | | | |  | | | | |  | | |  | | | |
| cOR: crude odds ratio; aOR: adjusted odds ratio; CI: confidence interval. | | | | | | | | | | | | | | | | | | | | | | | | | | | | | | | | | |  | |  | | | | |  | | | | |  | | |  | | | |
|  | | | | | | | | | |  | | | |  | | |  | | |  | | | | | |  | | | | | | | |  | |  | | | | |  | | | | |  | | |  | | | |
| All crude and adjusted odds ratios were statistically significant (p < 0.05).  Reference category: Children without a family history of the specified allergic disease. | | | | | | | | | | | | | | | | | | | | | | | | | | | | | | | | | |  | |  | | | | |  | | | | |  | | |  | | | |
| Adjusted odds ratios were estimated using logistic regression models adjusted for sex, school grade, population density category, and respiratory infections before the age of two years. | | | | | | | | | | | | | | | | | | | | | | | | | | | | | | | | | | | | | | | | | | | | | | | | | | | | |
| † Major allergy indicates the presence of bronchial asthma, atopic dermatitis, allergic rhinitis, or hives in any first-degree family member (father, mother, or siblings). | | | | | | | | | | | | | | | | | | | | | | | | | | | | | | | | | | | | | | | | | | | | | | | | |  | | | |
|  | | | | | | | | | | | | | | | | | | | | | | | | | | | | | | | | | | | | | | | |  | | | | | | | | | | | | |
| supplement Table 4-2. Family History of Allergies and Prevalence of Allergic Diseases (1992) | | | | | | | | | | | | | | | | | | | | | | | | | | | | | | |  | | | | | |  | | | | | | | |  | | |  | |  | | |
| Family History | | | | | n | | | | | | | | odds ratio | | | | | | Bronchial Asthma | | | | | | Atopic Dermatitis | | | | Allergic Rhinitis | | | Allergic Conjunctivitis | | | | | | | Japanese Cedar Pollinosis | | | | | Current Allergic Disease | | | |  | | | | |
| Major Allergy^†^ | | | + | | 26,783 | | | | | | | | cOR (95%CI) | | | | | | 2.92 (2.63-3.24) | | | | | | 2.56 (2.42-2.70) | | | | 2.41 (2.28-2.55) | | | 2.30 (2.12-2.50) | | | | | | | 2.63 (2.33-2.95) | | | | | 2.57 (2.46-2.68) | | | |  | | | | |
|  |  |  | - | | 19,935 | | | | | | | | aOR (95%CI) | | | | | | 2.71 (2.43-3.01) | | | | | | 2.51 (2.37-2.65) | | | | 2.39 (2.26-2.53) | | | 2.23 (2.05-2.43) | | | | | | | 2.59 (2.30-2.91) | | | | | 2.52 (2.42-2.64) | | | |  | | | | |
| Bronchial Asthma | | | + | | 7,637 | | | | | | | | cOR (95%CI) | | | | | | 4.16 (3.81-4.54) | | | | | | 1.69 (1.60-1.80) | | | | 1.67 (1.57-1.77) | | | 1.84 (1.69-2.00) | | | | | | | 1.61 (1.43-1.80) | | | | | 1.84 (1.75-1.94) | | | |  | | | | |
|  |  |  | - | | 39,081 | | | | | | | | aOR (95%CI) | | | | | | 3.84 (3.51-4.20) | | | | | | 1.64 (1.54-1.74) | | | | 1.62 (1.52-1.72) | | | 1.75 (1.61-1.91) | | | | | | | 1.55 (1.38-1.74) | | | | | 1.78 (1.69-1.87) | | | |  | | | | |
| Atopic Dermatitis | | | + | | 12,616 | | | | | | | | cOR (95%CI) | | | | | | 1.90 (1.74-2.07) | | | | | | 2.66 (2.53-2.79) | | | | 1.61 (1.52-1.69) | | | 1.65 (1.53-1.78) | | | | | | | 1.60 (1.44-1.76) | | | | | 2.07 (1.99-2.17) | | | |  | | | | |
|  |  |  | - | | 34,102 | | | | | | | | aOR (95%CI) | | | | | | 1.80 (1.65-1.97) | | | | | | 2.61 (2.49-2.75) | | | | 1.61 (1.53-1.70) | | | 1.61 (1.49-1.74) | | | | | | | 1.59 (1.44-1.76) | | | | | 2.06 (1.97-2.15) | | | |  | | | | |
| Allergic Rhinitis | | | + | | 15,504 | | | | | | | | cOR (95%CI) | | | | | | 2.05 (1.88-2.23) | | | | | | 1.80 (1.72-1.89) | | | | 2.70 (2.57-2.84) | | | 2.43 (2.26-2.61) | | | | | | | 2.96 (2.68-3.26) | | | | | 2.24 (2.15-2.34) | | | |  | | | | |
|  |  |  | - | | 31,214 | | | | | | | | aOR (95%CI) | | | | | | 1.93 (1.77-2.10) | | | | | | 1.76 (1.68-1.85) | | | | 2.69 (2.56-2.83) | | | 2.36 (2.20-2.54) | | | | | | | 2.93 (2.65-3.23) | | | | | 2.20 (2.11-2.29) | | | |  | | | | |
| Phase II n = 46,718 | | | |  | | | |  | | | | | | | |  | | | | | |  | | | | | |  | | |  | | | | | |  | | | | | | | |  | | |  | |  | | |
| cOR: crude odds ratio; aOR: adjusted odds ratio; CI: confidence interval. | | | | | | | | | | | | | | | | | | | | | | | | | | | |  | | |  | | | | | |  | | | | | | | |  | | |  | |  | | |
|  | | | | | |  | | |  | | | |  | | |  | | | | | |  | | | | | |  | | |  | | | | | |  | | | | | | | |  | | |  | |  | | |
| All crude and adjusted odds ratios were statistically significant (p < 0.05).  Reference category: Children without a family history of the specified allergic disease. | | | | | | | | | | | | | | | | | | | | | | | | | | | |  | | |  | | | | | |  | | | | | | | |  | | |  | |  | | |
| Adjusted odds ratios were estimated using logistic regression models adjusted for sex, school grade, population density category, and respiratory infections before the age of two years. | | | | | | | | | | | | | | | | | | | | | | | | | | | | | | | | | | | | | | | | | | | | | | | | | |  | | |
| † Major allergy indicates the presence of bronchial asthma, atopic dermatitis, allergic rhinitis, or hives in any first-degree family member (father, mother, or siblings). | | | | | | | | | | | | | | | | | | | | | | | | | | | | | | | | | | | | | | | | | | | | | | | |  | |  | | |
|  | supplement Table 4-3. Family History of Allergies and Prevalence of Allergic Diseases (2002) | | | | | | | | | | | | | | | | | | | | | | | | | | | | | | |  | | | | | | | | | |  | | | | |  | | | |  |  |
|  | Family History | | | | n | | | | | | | odds ratio | | | | | | | Bronchial Asthma | | | | | | Atopic Dermatitis | | | | Allergic Rhinitis | | | Allergic Conjunctivitis | | | | | | Japanese Cedar Pollinosis | | | | | Current Allergic Disease | | | |  | | | | | |
|  | Major Allergy^†^ | | + | | 23,724 | | | | | | | cOR (95%CI) | | | | | | | 3.04 (2.71-3.41) | | | | | | 2.74 (2.54-2.96) | | | | 2.89 (2.71-3.09) | | | 2.55 (2.33-2.79) | | | | | | 2.84 (2.52-3.21) | | | | | 2.90 (2.75-3.05) | | | |  | | | | | |
|  |  |  | - | | 12,504 | | | | | | | aOR (95%CI) | | | | | | | 2.82 (2.51-3.17) | | | | | | 2.68 (2.48-2.90) | | | | 2.86 (2.68-3.05) | | | 2.48 (2.26-2.71) | | | | | | 2.77 (2.46-3.13) | | | | | 2.85 (2.70-3.00) | | | |  | | | | | |
|  | Bronchial Asthma | | + | | 8,784 | | | | | | | cOR (95%CI) | | | | | | | 3.92 (3.60-4.26) | | | | | | 1.61 (1.51-1.72) | | | | 1.64 (1.55-1.74) | | | 1.60 (1.49-1.73) | | | | | | 1.49 (1.35-1.64) | | | | | 1.84 (1.76-1.94) | | | |  | | | | | |
|  |  |  | - | | 27,444 | | | | | | | aOR (95%CI) | | | | | | | 3.64 (3.34-3.97) | | | | | | 1.57 (1.47-1.67) | | | | 1.61 (1.52-1.71) | | | 1.56 (1.44-1.68) | | | | | | 1.45 (1.31-1.59) | | | | | 1.79 (1.71-1.89) | | | |  | | | | | |
|  | Atopic Dermatitis | | + | | 9,447 | | | | | | | cOR (95%CI) | | | | | | | 2.04 (1.87-2.22) | | | | | | 3.42 (3.21-3.63) | | | | 1.62 (1.53-1.71) | | | 1.74 (1.62-1.87) | | | | | | 1.60 (1.46-1.75) | | | | | 2.16 (2.06-2.27) | | | |  | | | | | |
|  |  |  | - | | 26,781 | | | | | | | aOR (95%CI) | | | | | | | 1.92 (1.76-2.10) | | | | | | 3.36 (3.16-3.57) | | | | 1.58 (1.49-1.67) | | | 1.68 (1.56-1.81) | | | | | | 1.55 (1.41-1.70) | | | | | 2.12 (2.02-2.22) | | | |  | | | | | |
|  | Allergic Rhinitis | | + | | 16,365 | | | | | | | cOR (95%CI) | | | | | | | 1.96 (1.80-2.14) | | | | | | 1.79 (1.68-1.90) | | | | 3.11 (2.94-3.28) | | | 2.49 (2.31-2.67) | | | | | | 3.25 (2.95-3.59) | | | | | 2.52 (2.41-2.63) | | | |  | | | | | |
|  |  |  | - | | 19,863 | | | | | | | aOR (95%CI) | | | | | | | 1.85 (1.70-2.02) | | | | | | 1.75 (1.65-1.86) | | | | 3.10 (2.93-3.27) | | | 2.42 (2.25-2.61) | | | | | | 3.20 (2.90-3.53) | | | | | 2.48 (2.38-2.60) | | | |  | | | | | |
|  | Phase III n = 36,228 | | | | | | | | |  | | | | |  | | |  | | | | | |  | | | | | |  | |  | | | | | | | | | |  | | | | |  | | | |  |  |
|  | cOR: crude odds ratio; aOR: adjusted odds ratio; CI: confidence interval. | | | | | | | | | | | | | | | | | | | | | | | | | | | | |  | |  | | | | | | | | | |  | | | | |  | | | |  |  |
|  |  | | | | | |  | | | |  | | | |  | | |  | | | | | |  | | | | | |  | |  | | | | | | | | | |  | | | | |  | | | |  |  |
|  | All crude and adjusted odds ratios were statistically significant (p < 0.05).  Reference category: Children without a family history of the specified allergic disease. | | | | | | | | | | | | | | | | | | | | | | | | | | | | |  | |  | | | | | | | | | |  | | | | |  | | | |  |  |
|  | Adjusted odds ratios were estimated using logistic regression models adjusted for sex, school grade, population density category, and respiratory infections before the age of two years. | | | | | | | | | | | | | | | | | | | | | | | | | | | | | | | | | | | | | | | | | | | | | |  | | | | | |
|  | † Major allergy indicates the presence of bronchial asthma, atopic dermatitis, allergic rhinitis, or hives in any first-degree family member (father, mother, or siblings). | | | | | | | | | | | | | | | | | | | | | | | | | | | | | | | | | | | | | | | | | | | | | | | | | |  |  |

| supplement Table 4-4 Family History of Allergies and Prevalence of Allergic Diseases (2012) | | | | | | | | | | | | | | |  | | | | | |
| --- | --- | --- | --- | --- | --- | --- | --- | --- | --- | --- | --- | --- | --- | --- | --- | --- | --- | --- | --- | --- |
|  | | | | |  | | |  |  | |  | | |  |  | | | | | |
|  | Family History | | n | | | | odds ratio | | | Bronchial Asthma | | Atopic Dermatitis | Allergic Rhinitis | | | Allergic Conjunctivitis | Japanese Cedar Pollinosis | Current Allergic Disease | |  |
|  | Major Allergy^†^ | + | 23,126 | | | | cOR (95%CI) | | | 4.11 (3.48-4.86) | | 3.13 (2.85-3.45) | 3.14 (2.95-3.34) | | | 2.82 (2.57-3.09) | 2.87 (2.59-3.18) | 3.24 (3.07-3.43) | |  |
|  |  | - | 10,776 | | | | aOR (95%CI) | | | 3.67 (3.10-4.34) | | 3.05 (2.77-3.36) | 3.18 (2.99-3.39) | | | 2.77 (2.52-3.04) | 2.86 (2.58-3.17) | 3.25 (3.07-3.43) | |  |
|  | Bronchial Asthma | + | 9,777 | | | | cOR (95%CI) | | | 4.87 (4.38-5.41) | | 1.74 (1.63-1.87) | 1.68 (1.59-1.76) | | | 1.59 (1.49-1.71) | 1.33 (1.23-1.44) | 1.86 (1.77-1.95) | |  |
|  |  | - | 24,125 | | | | aOR (95%CI) | | | 4.25 (3.82-4.73) | | 1.68 (1.57-1.80) | 1.65 (1.57-1.74) | | | 1.53 (1.43-1.65) | 1.30 (1.20-1.40) | 1.81 (1.72-1.90) | |  |
|  | Atopic Dermatitis | + | 8,550 | | | | cOR (95%CI) | | | 2.27 (2.05-2.51) | | 3.84 (3.59-4.11) | 1.68 (1.59-1.77) | | | 1.82 (1.70-1.96) | 1.64 (1.52-1.77) | 2.24 (2.13-2.36) | |  |
|  |  | - | 25,352 | | | | aOR (95%CI) | | | 2.12 (1.91-2.35) | | 3.78 (3.53-4.04) | 1.68 (1.59-1.77) | | | 1.80 (1.67-1.93) | 1.63 (1.51-1.76) | 2.24 (2.13-2.36) | |  |
|  | Allergic Rhinitis | + | 17,724 | | | | cOR (95%CI) | | | 2.42 (2.16-2.70) | | 1.95 (1.82-2.09) | 3.35 (3.18-3.53) | | | 2.76 (2.56-2.97) | 3.09 (2.85-3.36) | 2.96 (2.82-3.10) | |  |
|  |  | - | 16,178 | | | | aOR (95%CI) | | | 2.23 (1.99-2.50) | | 1.91 (1.78-2.05) | 3.41 (3.23-3.59) | | | 2.71 (2.52-2.92) | 3.08 (2.84-3.35) | 2.96 (2.83-3.10) | |  |
| Phase IV n = 33,902  cOR: crude odds ratio; aOR: adjusted odds ratio; CI: confidence interval. | | | | | | | | | | | | | |  |  | | | | | |
|  | | | |  | |  | |  |  | |  | | |  |  | | | | | |
| All crude and adjusted odds ratios were statistically significant (p < 0.05).  Reference category: Children without a family history of the specified allergic disease. | | | | | | | | | | | | | |  |  | | | | | |
| Adjusted odds ratios were estimated using logistic regression models adjusted for sex, school grade, population density category, and respiratory infections before the age of two years. | | | | | | | | | | | | | | | | | | |  | |
| † Major allergy indicates the presence of bronchial asthma, atopic dermatitis, allergic rhinitis, or hives in any first-degree family member (father, mother, or siblings). | | | | | | | | | | | | | | | | | | | | |

| supplement Table 4-5. Family History of Allergies and Prevalence of Allergic Diseases (2022) | | | | | | | | | | | | | | |  | |  | |  |  |
| --- | --- | --- | --- | --- | --- | --- | --- | --- | --- | --- | --- | --- | --- | --- | --- | --- | --- | --- | --- | --- |
| Family History | | n | | | odds ratio | | | Bronchial Asthma | Atopic Dermatitis | | Allergic Rhinitis | | Allergic Conjunctivitis | Japanese Cedar Pollinosis | | Current Allergic Disease | |  | | |
| Major Allergy^†^ | + | 21,821 | | | cOR (95%CI) | | | 4.05 (3.13-5.25) | 2.88 (2.60-3.19) | | 3.20 (2.99-3.41) | | 2.66 (2.40-2.95) | 2.45 (2.23-2.69) | | 3.14 (2.96-3.34) | |  | | |
|  | - | 7,732 | | | aOR (95%CI) | | | 3.54 (2.72-4.60) | 2.83 (2.55-3.14) | | 3.20 (2.99-3.42) | | 2.59 (2.33-2.87) | 2.43 (2.22-2.67) | | 3.11 (2.93-3.31) | |  | | |
| Bronchial Asthma | + | 8,673 | | | cOR (95%CI) | | | 5.40 (4.64-6.28) | 1.65 (1.53-1.77) | | 1.53 (1.45-1.61) | | 1.51 (1.40-1.62) | 1.24 (1.16-1.33) | | 1.65 (1.57-1.74) | |  | | |
|  | - | 20,880 | | | aOR (95%CI) | | | 4.56 (3.91-5.32) | 1.60 (1.49-1.72) | | 1.51 (1.43-1.59) | | 1.45 (1.35-1.56) | 1.22 (1.14-1.31) | | 1.61 (1.53-1.70) | |  | | |
| Atopic Dermatitis | + | 8,701 | | | cOR (95%CI) | | | 1.93 (1.67-2.22) | 3.95 (3.69-4.24) | | 1.57 (1.49-1.65) | | 1.69 (1.57-1.81) | 1.56 (1.45-1.67) | | 2.04 (1.93-2.15) | |  | | |
|  | - | 20,852 | | | aOR (95%CI) | | | 1.77 (1.53-2.04) | 3.91 (3.64-4.19) | | 1.56 (1.48-1.65) | | 1.66 (1.55-1.79) | 1.55 (1.45-1.66) | | 2.03 (1.93-2.14) | |  | | |
| Allergic Rhinitis | + | 17,423 | | | cOR (95%CI) | | | 2.22 (1.88-2.61) | 1.78 (1.65-1.91) | | 3.34 (3.16-3.53) | | 2.54 (2.34-2.75) | 2.51 (2.33-2.71) | | 2.85 (2.71-3.00) | |  | | |
|  | - | 12,130 | | | aOR (95%CI) | | | 1.97 (1.67-2.32) | 1.75 (1.62-1.88) | | 3.36 (3.18-3.55) | | 2.48 (2.29-2.70) | 2.50 (2.32-2.69) | | 2.82 (2.68-2.97) | |  | | |
|  |  |  | | |  | | | | |  | |  | | |  | |  | |  |  |
| Phase V n = 29.553  cOR: crude odds ratio; aOR: adjusted odds ratio; CI: confidence interval. | | | | | | | | | | | |  | | |  | |  | |  |  |
|  | | |  |  | |  |  | | |  | |  | | |  | |  | |  |  |
| All crude and adjusted odds ratios were statistically significant (p < 0.05).  Reference category: Children without a family history of the specified allergic disease. | | | | | | | | | | | |  | | |  | |  | |  |  |
| Adjusted odds ratios were estimated using logistic regression models adjusted for sex, school grade, population density category, and respiratory infections before the age of two years. | | | | | | | | | | | | | | | | | | | | |
| † Major allergy indicates the presence of bronchial asthma, atopic dermatitis, allergic rhinitis, or hives in any first-degree family member (father, mother, or siblings). | | | | | | | | | | | | | | | | | | | |  |
